# Potential Shifts in the Oral Microbiome Induced by Bariatric Surgery—A Scoping Review

**DOI:** 10.3390/antibiotics14070695

**Published:** 2025-07-10

**Authors:** Zuzanna Ślebioda, Hélène Rangé, Marta Strózik-Wieczorek, Marzena Liliana Wyganowska

**Affiliations:** 1 Department of Periodontology and Oral Mucosa Diseases, Poznan University of Medical Sciences, 61-701 Poznan, Poland; klchstomiper@ump.edu.pl; 2 Department of Periodontology, Faculty of Odontology, CHU Rennes, University of Rennes, 35000 Rennes, France; helene.range@univ-rennes.fr; 3 CIC 1414 (Centre d’Investigation Clinique de Rennes), CHU Rennes, University of Rennes, Inserm, 35000 Rennes, France; 4 UMR S 1317 Institut Numecan (Nutrition, Métabolismes et Cancer), 35000 Rennes, France; 5 Denta Private Dental Practice, 53-022 Wroclaw, Poland

**Keywords:** bariatric surgery, oral microbiome, periodontology, oral bacteria

## Abstract

**Background**: The oral microbiome differs in obese patients compared to normal-weight subjects. Microbiologic shifts very often appear after surgical interventions such as bariatric surgery (BS) and in immunocompromised patients. However, the oral microbiome composition and load in subjects after bariatric surgery are unclear. **Aim**: The aim of this review is to summarize the current state of the art related to the oral microbiome shift induced by bariatric surgery and to discuss its implications on oral cavity health. **Methods**: Electronic databases: PubMed/Medline, Web of Science, and Cochrane Library were searched for articles published up to March 30, 2025, describing prospective studies focused on changes in the oral microbiota of patients who underwent bariatric surgery. **Results**: Eight studies measuring the oral microbiome with different approaches—16S ribosomal RNA (16S rRNA) sequencing, polymerase chain reaction (PCR), culture, and matrix-assisted laser desorption ionization time of flight mass spectrometry (MALDI TOF MS)—were included in this review. The following bariatric techniques were used: sleeve gastrectomy, Roux-en-Y gastric bypass, Omega loop gastric bypass, and laparoscopic gastric plication. The follow-up period ranged from 3 to 12 months. The results of microbiologic studies were unequivocal. There was an increment in *Streptococcus mutans* reported, high levels of *Candida* species, and increased rates of some periodontitis-associated bacteria (*Porphyromonas gingivalis*) in the post-bariatric surgery period, though some studies suggested a shift towards non-pathogenic composition of the oral microbiome in prospective observations. **Conclusions**: The local oral microbial homeostasis becomes strongly impacted by the bariatric surgical treatment itself as well as its consequences in the further post-operative period. Therefore, obese patients undergoing BS require very careful dental observation.

## 1. Introduction

According to the World Health Organization, there is an increase in obese people with a body mass index (BMI) over 30 kg/m^2^ in both developed and developing countries. Worldwide, more than one-third of adults show a BMI of >25 kg/m^2^, which classifies them as overweight or obese. Obesity, defined simply as excess body weight for height, is in fact a complex, multifactorial disease, with genetic, behavioral, socioeconomic, and environmental backgrounds [1,2]. Also, among children and adolescents, the mean BMI and prevalence of obesity increased in the last decades. Although it has plateaued at high levels in many high-income countries since around 2000, it has accelerated in East, South, and Southeast Asia [1,2,3,4].

Obesity correlates with an increased risk of cardiovascular diseases, type 2 diabetes, depression, several types of cancers, and an increased risk of debilitating morbidity and mortality, which makes it a serious public health problem [2,5,6].

In recent years, the role of gut microbiome composition in obesity development has raised broad attention [6,7]. By enhancing gut–brain-mediated fat intake and hunger signaling, and by providing an extra energy source, several common gut microbes may promote obesity [8,9]. The differences in microbiome constitution between obese and normal-weight patients were described in recent studies at various levels of the gastrointestinal tract, including the oral cavity [9,10,11,12]. The data from Duvallet et al.’s meta-analysis shows that the obesity-associated species largely differ between studies [13]. Many studies indicate a link between body weight and certain oral diseases [14,15,16]. The intensity of the cytokines’ secretion corresponds to the amount of fat present. Therefore, obesity may alter the response of the host to the antigens derived from bacterial plaque and thus cause disturbances in the inflammatory response [17]. It was shown that excessive adipose tissue accumulation also promotes inflammation in periodontal tissues. The response to infection with periodontal pathogens is greater in obese people than in the average population [18].

It may be expected that microbiome shifts would also appear in the prospective observation of obese patients who reduce their body weight using various therapeutic approaches, including dietary, pharmacologic, and surgical methods.

By limiting the intake of food and interfering with digestion, BS is considered one of the most effective strategies in body weight reduction [19]. Despite the direct effect on body weight loss, this treatment approach influences the health condition on the systemic level, occupying various, not yet clearly understood mechanisms. Those involve alterations in the levels of intestinal hormones, including glucagon-like peptide 1 (GLP-1), adiponectin, ghrelin, and leptin. Post-bariatric intestinal microbiome shifts have been reported in several studies, while salivary microbiome composition has not been evaluated extensively so far [6,20,21,22,23,24,25,26,27,28,29,30].

The aim of this review is to summarize the current state of the art related to the oral microbiome shift induced by bariatric surgery and to discuss its implications on oral cavity health.

## 2. Methods

The exposure of interest for the current study was any form of BS, irrespective of the type of surgery and follow-up period. The outcome was the change in oral microbiota after BS. Therefore, we included prospective studies, excluding observational and cross-sectional analyses. The focused question was whether any changes occurred in the oral microbiota of patients who underwent BS in the post-surgical period.

Electronic databases: PubMed/Medline, Web of Science, and Cochrane Library were searched for articles published up to 30 March 2025, with different Medical Subject Headings (MeSH) and supplementary non-MeSH terms, including “bariatric surgery” and “oral microbiome” or “oral microbiota” or “oral bacteria”. A language restriction was implemented, and only the full-text articles in English were finally qualified for further evaluation. Additionally, a manual search of the potentially eligible references was performed. Initially, the records were assessed by two independent authors according to the relevance of the title and/or abstract. Studies considered potentially eligible by at least one of the reviewers in the initial search were then verified in their entirety by the same two authors. Further analysis included full texts describing the studies that met the following inclusion criteria: human clinical studies comparing changes in oral microbiota before and after BS in a prospective manner, irrespective of sex and age. The reviewers were not blinded to the authorship of the analyzed studies. The data from each study were extracted independently by the two reviewers and included authorship, year of publication, country of origin, type of intervention and comparisons, study population and design, follow-up period, microbiota investigation technique, location of specimen collection, and outcomes (changes in levels of the oral microbiome), which were introduced into an Excel spreadsheet for further analysis. The search strategy is provided in the Supplementary Materials (Supplement S1).

Guidelines given by the Preferred Reporting Items for Systematic Reviews and Meta-analyses extension for Scoping Reviews (PRISMA-ScR) were used for the framework designing, application, and reporting of the current systematic review. The protocol was registered with the International Prospective Register of Systematic Reviews and was assigned the following identification code: PROSPERO CRD420251042284.

## 3. Results

### 3.1. Sources Identification

**Figure 1:**
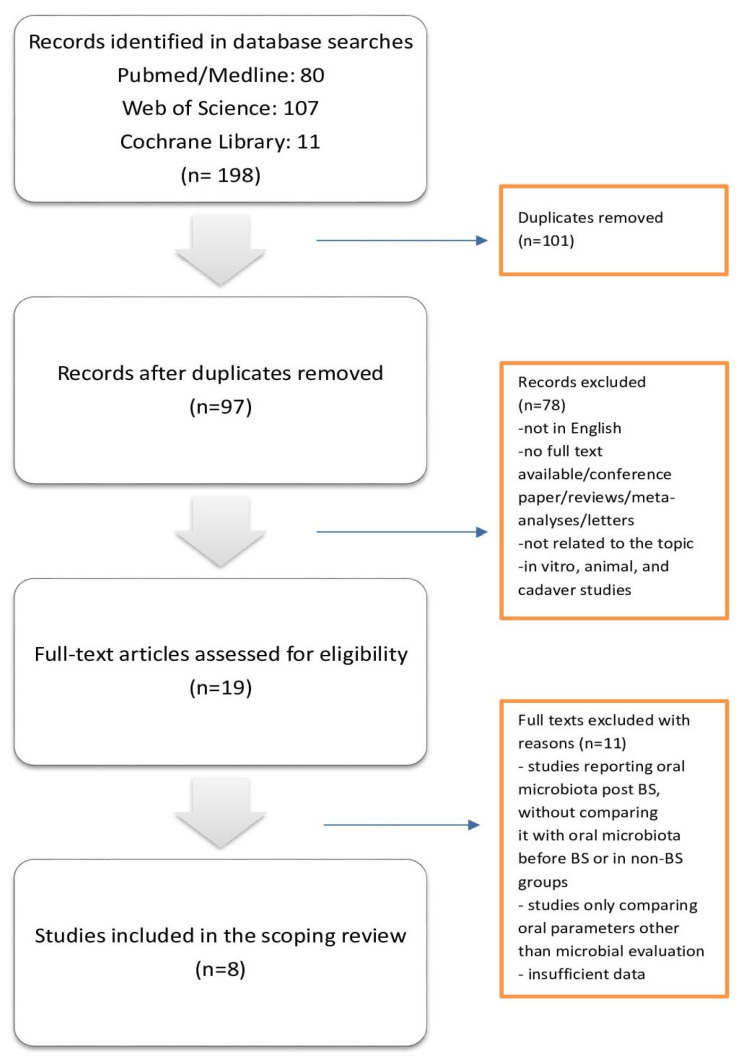
Search strategy according to PRISMA guidelines.

Figure 1 illustrates the flowchart of the article inclusion strategy based on the PRISMA guidelines. In the initial search, 198 articles were identified. After duplicate removal (101), screening of the 97 titles and abstracts was performed to exclude irrelevant articles. Nineteen full-text articles were reviewed, and a manual search of references for these articles was performed, not revealing any more relevant papers. Finally, eight articles were included in this review.

### 3.2. Study Characteristics

We included eight studies that investigated the changes in oral microbiota after BS: three from Brazil and one each from the Czech Republic, Hungary, Korea, Poland, and the USA. Bariatric techniques used included sleeve gastrectomy, Roux-en-Y gastric bypass, Omega loop gastric bypass, and laparoscopic gastric plication. The exact type of gastric bypass applied in Balogh et al.’s study was not defined [28]. The follow-up period ranged from 12 weeks in Shillitoe et al.’s study [22] to 12 months [6,25,28]. In the remaining four reports, the observation period was 6 months [23,24,26,27]. In all of the studies, the changes in oral microbiota were compared between the moment before the surgery and in predesigned follow-up periods (3, 6, or 12 months) in the same selected participants. Additionally, in two studies, microbiologic composition was compared between BS patients and those who had not undergone BS [27,28]. Microbiologic specimens were isolated from crevicular fluid (two studies; [25,28]), saliva (five studies; [6,22,23,24,26]), subgingival plaque (one study; [23]), and oral swabs (two studies; [23,27]). In four studies, the 16S rRNA gene sequence analysis technique was utilized [6,23,26,27], two used the real-time quantitative polymerase chain reaction (RT-qPCR) technique for relative DNA quantification of specific microbial targets [22,25], one expressed microbiological counts as colony-forming units per milliliter (CFU/mL saliva) on selective culture media [24], while another one used matrix-assisted laser desorption ionization time of flight mass spectrometry (MALDI-TOF MS) for identification [28].

Table 1 shows the main characteristics of the studies included in this review and the most essential conclusions.

### 3.3. Results of the Studies

As presented in Table 1, which summarizes the study sample sizes, microbiological methods used, and key microbial shifts observed post-surgery, there was heterogeneity in the approach to oral microbiota identification. Based on the reported data, it is difficult to indicate one evident trend of microbial shift in bariatric patients. Moreover, changes in oral microbiota were highly heterogeneous between individuals within each study.

A significant increase in salivary *Streptococcus mutans* following BS was reported in the study by Hashizume et al. [24]. Another species of *Streptococci* that tended to increase after BS was *Streptococcus salivarius* [23]. *Streptococcus oralis* had a positive correlation with BMI in the study by Dzunkova et al. [6]. Hashizume et al. also reported elevated levels of *Candida albicans* in the study population, though no significant shift was observed after BS [24]. Meanwhile, in the study by Balogh et al., *Candida albicans* and *non-albicans Candida species*—*C. dubliniensis*, *C. kefyr*, and *C. lusitaniae*—emerged after surgery, both in terms of the proportion of subjects and a significant germ count. At the same time, the proportion of patients with *Neisseria* decreased significantly after BS. The growth of *Candida* was observed only where *Neisseria* was absent throughout or eliminated after surgery. Other genera found in a considerably increased proportion of subjects upon weight loss in their study included *Prevotella*, though the difference before and after BS was not statistically significant [28]. *Prevotella intermedia* count, among the four other pathogens, was also measured in the study by Sales-Peres et al. However, the key finding of their research was that the amount of *Porphyromonas gingivalis* significantly increased after BS, which correlated with the progression of periodontitis [25]. In their study, four periodontal pathogens (*Porphyromonas gingivalis*, *Tannerella forsythia*, *Treponema denticola*, and *Prevotella intermedia*) were evaluated in the crevicular fluid. There was a six-fold increment in the amount of *P. gingivalis* after 6 months from BS. The boost in *P. gingivalis* during the 12-month follow-up was associated with the probing pocket depth and gingival bleeding. The fluctuations in the amount of the remaining three pathogens evaluated in the oral cavity in this study (*T. forsythia*, *T. denticola*, and *P. intermedia*) were less spectacular and considered statistically nonsignificant. There was an initial increase during the first period of the observation followed by a decrease in the amount of those bacteria in the final endpoint (12 months after BS). A time–group interaction effect was observed for the periodontitis-associated bacteria *Prevotella nigrescens* and *Porphyromonas endodontalis*, with a decrease in relative abundance over time in the control group but an increase in the gastroplasty group in Ribeiro et al.’s study [26]. The caries activity increased in both study groups, while the periodontal status worsened in the gastroplasty group in 3 months post-BS. The results of their study also showed a significant time effect for *Neisseria subfava* (increase in the abundance), which contrasts with the previously cited study by Balogh et al., which showed a decrement in *Neisseria* [28]. Meanwhile, in the study by Kim et al., there was a shift towards the expansion of microbiomes associated with a healthy state, which increased over time (*Streptococcus salivarius* and various *Veillonella* spp.). The clusters containing periodontal pathogens, including *Porphyromonas* spp., tended to diminish during subsequent follow-ups [23]. A significant post-surgical increase in salivary *Bifidobacteria* species, especially in patients with concomitant diabetes mellitus, was observed in the study by Shillitoe et al. [22]; *Bacteroidetes* increased in abundance in the oral cavity 6 months after BS in Stefura et al.’s study. Patients with less favorable outcomes in terms of body weight loss presented an increase in the phylum *Fusobacteria* and a decrease in the phylum *Firmicutes* in the oral cavity [27].

The key microbiological findings in the oral cavity related to the post-bariatric surgery period were increases in *Candida albicans* and *non-albicans* count; elimination of *Neisseria*; increased proportions of *Veillonella* and *Megasphaera micronuciformis* with the reduction in BMI; increased salivary levels of *Streptococcus mutans*; and the increment of microbiomes associated with a healthy state, including *Streptococcus salivarius*. Clusters containing periodontal pathogens, including *Porphyromonas* spp., tended to diminish in one study, while another study reported an increment in *Porphyromonas gingivalis* count after BS. *Bifidobacteria, Bacteroidetes*, and Fusobacteria counts increased, while Firmicutes levels decreased in the post-bariatric period.

**Figure 2:**
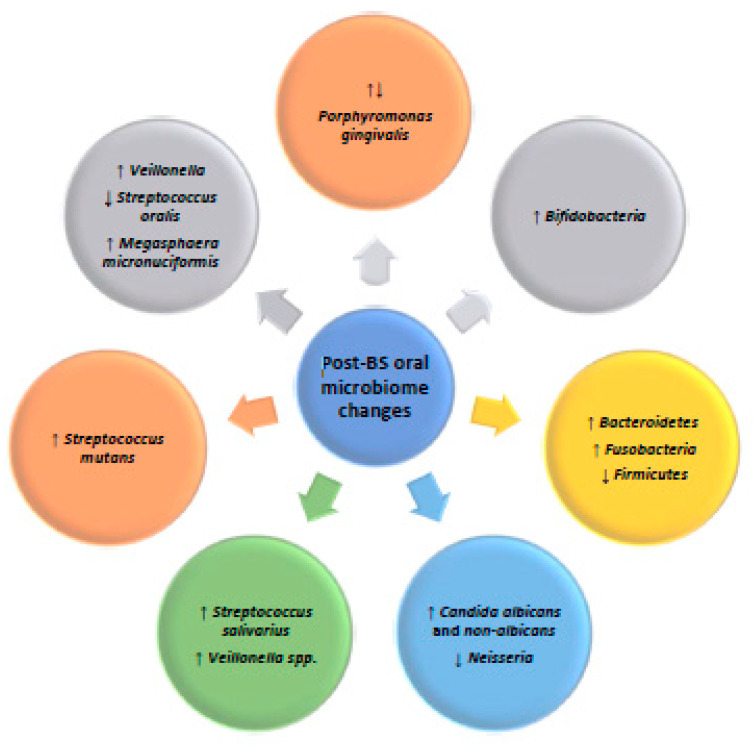
Oral microbiome changes that were observed in the post-bariatric surgery period (↑—increase in the number; ↓—decrease in the number).

Figure 2 depicts the most relevant trends in the oral microbiome changes that occurred after BS.

## 4. Discussion

Significant changes in the oral cavity following bariatric surgery were in the past described as associated with a more frequent intake of small amounts of food, gastroesophageal reflux, and vomiting [31]. This leads to a decrease in both resting and stimulated salivary flow, reduced salivary pH and buffering capacity, and lower concentrations of minerals in saliva [32,33], which increase the risk of dental caries and alter the oral microbiome [34,35,36].

Apart from being obese, patients who qualify for bariatric surgical treatment very often present with several other systemic co-morbidities resulting from chronic overweight. Psychological and social impacts of obesity include a risk of depression and anxiety, sometimes leading to social isolation [37,38]. Such a complex health situation may obviously influence the local and systemic microbiologic homeostasis. For example, some recent observations indicate that the salivary microbiome of patients with obesity is dominated by *Streptococcus* species with a great potential for carbohydrate metabolism [11,12]. This could result from the acidification of the oral environment, promoting the growth of obesity-associated bacterial species. The variability in salivary buffer capacity in obesity-related type 2 diabetes is induced by hyperglycemia and elevated salivary glucose levels. Meanwhile, the altered salivary microbiome may interfere with taste preferences, quantity and quality of food consumption, and liquid intake [39]. Obesity was associated with a higher risk of periodontitis in Jagannathachary and Kamaraj’s study [40], Nascimento et al.’s study [41], and Suvan et al.’s study [42].

However, in this review, we aimed to observe microbiologic changes induced not strictly by obesity and its complications but resulting from bariatric treatment, in most cases followed by a rapid weight loss. That makes the study population very heterogeneous, showing a complex general health state. The results of the presented studies are unambiguous, which hinders stating very definite conclusions at this point. This stands in line with the observations of Adawi et al. shown in their recent systematic review [43].

When considering the studies related to the impact of BS on the condition of periodontal tissues and periodontitis-associated bacteria, there were two opposite trends presented. The main hypothesis of Sales-Peres et al.’s study was based on the assumption that gastric bypass surgery reduces the inflammatory response in the body. That, together with a decrease in the presence of periodontitis-associated bacteria, could result in an improvement in the periodontal status after 12 months post-surgery. To verify this hypothesis, the levels of four pathogens relevant in the development of periodontitis, namely *P. gingivalis*, *T. forsythia*, *T. denticola*, and *P. intermedia*, were evaluated in the gingival fluid in patients treated with BS. Surprisingly, the amount of *P. gingivalis* was six times higher 6 months after the surgery. The presence of *P. gingivalis* increased during the follow-up and was associated with the probing pocket depth and the gingival bleeding index after a year post-BS [25]. High quantities of *P. gingivalis* in the postoperative group were also found in a cross-sectional study by Pataro et al., who were also astonished by this finding, since weight reduction decreases the inflammatory response [44]. It is well known that low pH inhibits the growth of *P. gingivalis*, reducing its viability and the expression of virulence factors (gingipains, fimbriae). However, it promotes the development of cariogenic bacteria, such as *Streptococcus mutans* [45]. *P. gingivalis* does exhibit some adaptive abilities to pH changes, for instance, by producing enzymes that neutralize the environment (e.g., arginine metabolic pathways) as well as by interacting with other microorganisms that help buffer the surroundings [46]. A low pH significantly limits the motility of spirochetes and their ability to penetrate the epithelium, resulting in reduced activity of *Treponema denticola* [47]. Meanwhile, although the number of patients with the *T. forsythia* and *T. denticola* bacteria increased slightly during the follow-up, their relative quantity in Sales-Peres et al.’s study decreased. This does not correspond with the progression of periodontal disease in their study population, typically related to the increment in the quantities of red complex bacteria [25]. In the study by Ribeiro et al. [26], the authors also hypothesized that as both obesity and periodontitis are inflammatory diseases, the decrease in inflammation (as evidenced by the reduction in salivary IFNγ and IL6 levels) could lead to improvement in periodontal status. Like in the study by Sales-Peres et al., a slight worsening in periodontitis was observed at 3 months in the gastroplasty group, while the control group showed a slight improvement in periodontal status [25]. This, however, worsened again at 6 months. According to the authors, this was possibly due to patients’ demotivation, concomitant with the stabilization of weight loss, and also profound changes in dietary and eating habits, which make the control of dental biofilm difficult [26]. This stands in line with the results of the systematic review and meta-analysis performed by Fontanille et al., who observed increased periodontal inflammation in the BS group compared to controls during the 6-month follow-up, but 12 months after baseline, the difference between the groups was no longer significant [48]. Contrary conclusions were derived in the study by Kim et al., who showed that the oral microbiome at 6 months post-bariatric surgery indicated a potential shift toward a healthy periodontal state. In their study, there were no significant differences in periodontal status between the groups. Although distinct species associated with periodontal disease were found in subgingival plaque in the obese, surgically treated subjects (*Filifactor alocis*, *Peptostreptococcaceae* spp., *Prevotella* spp., and *Treponema maltophilum*), microbiomes associated with a healthy state increased over time (*Streptococcus salivarius* and *various Veillonella* spp.), while the clusters, including *Porphyromonas* spp., related to the development of periodontitis, tended to diminish. The authors emphasized that weight loss interventions may positively impact oral microbial communities even in the absence of clinical periodontitis [23]. The periodontal condition did not deteriorate after BS in the study by Balogh et al., who found no signs of inflammation in any group, no attachment loss greater than 3 mm, and no pathologically deep periodontal pockets in subjects after BS and weight loss [28].

In the same study, the authors demonstrated that *Candida albicans* and *non-albicans Candida* species emerged after surgery, especially in the absence of *Neisseria*. A negative correlation with these two species was also demonstrated in an in vitro study by Janus et al. [49]. *C. albicans* shifts in patients undergoing BS were also evaluated in a study by Hashizume et al. They detected high *C. albicans* levels both before and after the intervention, so there was no direct correlation between the BS and the level of oral fungi [24]. Nevertheless, the observed levels were above the norm, which illustrates that obese patients are at risk of oral candidiasis, regardless of the treatment approach. As emphasized in Singh’s review, the pathogenicity of *Candida* and the incidence of candidiasis depend on the immune status of the host cell. A delicate balance between the fungi and the host’s immune status determines the commensal or parasitic relationship [50]. Most commonly, *non-albicans Candida* species develop in the oral cavities in immunosuppressed patients, for example, during chemotherapy or in HIV-infected individuals [28].

A study by Hashizume et al. also investigated patients’ saliva sample levels of *Streptococcus* and *Lactobacillus* spp. before and 6 months after bariatric surgery. An elevated *S. mutans* level 6 months after BS was found. Although in general the levels were not high, there was a significant difference between the pre- and post-surgical period, with a tendency to increase with time. The potential justification of this finding could be changes in dietary behavior following BS. On the one hand, often there is a reduced consumption of sugars and fats after BS; on the other hand, bad eating habits, such as high sugar intake, binge eating, and night-time eating, may persist after surgery and are often hidden by patients [24]. Furthermore, as a result of reduced stomach size and intestinal absorption following BS, meal patterns change, with an increased number of meals per day. The frequency of sugary food intake increases, which may explain the increase in the salivary levels of *S. mutans*. An interference of the medications used by patients after BS, like proton pump inhibitors and benzimidazoles, with the growth of bacteria should also be considered. However, based on the in vitro studies, those drugs inhibit *S. mutans*, so a reduction in the bacterial count should be expected [51]. In the presence of high concentrations of *S. mutans*, the risk of dental caries increases rapidly. In this study, however, the clinical correlation between bacteria and teeth status was not evaluated.

Due to a high risk of surgical wound infection in bariatric surgery, an antibiotic prophylaxis is strongly recommended [52]. Freeman et al. described 37 different antibiotic regimens used in the prevention of surgical site infections in bariatric surgery, indicating that although cefazolin is the most recommended drug, other options are widely used [53]. Systemic antibiotic therapy strongly influences the post-surgical composition of the gut microbiota, as demonstrated in Nalluri et al.’s study [54].

In this review, we were not able to compare the effects of peri-operational antibiotic treatment on the composition of the oral microbiome, as the data on this accessory treatment approach were not provided in most of the included papers. The authors described the types of surgical approaches rather than other additional pharmacological strategies, which were probably also utilized in most of the patients. Nevertheless, after defining more precisely the tendency of local oral microbial shifts, the next step could be to evaluate oral microbiota modifications induced by systemic antibiotics during bariatric surgical treatment. A better understanding of oral microbiologic changes in BS patients would also allow for defining the necessity of using local antibiotics to prevent deterioration of oral health, which was often observed in the studies presented in this review.

## 5. Limitations

There are some limitations of this review related to the heterogeneity in the approach to oral microbiota identification. Oral microbiota shifts were highly heterogeneous between individuals within each study. The number of studies included in this review was also limited. There was insufficient data on subjects’ oral hygiene maintenance and on monitoring or control of participants’ dietary intake, including medications and supplements. Since individual-specific factors significantly influence salivary microbiome composition, the absence of this information introduces considerable variability, potentially undermining the validity and interpretability of the results. Therefore, it was hard to demonstrate one evident tendency in the microbial shift in bariatric patients. Nevertheless, with caution, some conclusions can be stated.

## 6. Conclusions

The mechanisms of BS’s impact on oral microbiota and health seem to be very complex, modified by several local and systemic factors. Bariatric surgical interventions influence the salivary microbiota, but at this point, it is difficult to indicate one direction in the shift. There are observations indicating both positive and negative correlations between pre- and post-surgical oral microbial status with the clinical findings. However, the studies exploring the connection between microbiome modification and the evolution of oral lesions are scarce.

There is an urgent need to expand the research on potential correlations between microbiologic status and several oral pathologies affecting soft and hard oral tissues in obese patients undergoing BS. Nevertheless, it was definitely proven that the local oral microbial homeostasis becomes strongly impacted by the bariatric surgical treatment itself as well as its consequences in the further post-operative period. Therefore, obese patients undergoing BS require very careful dental and periodontal observation. We believe that further research on the oral implications of BS is required to develop an algorithm of oral care for patients qualified and undergoing this surgical therapy.

## Figures and Tables

**Table 1:** Characteristics of the studies included in the review.

Author/Year /Design	Country	Study Population (Number, M/F Ratio, Mean Age [Years])	Intervention	Follow-Up	Microbiologic Evaluation	Results
Balogh et al., 2020 Follow-up study [28]	Hungary	57 total, M/F 33/24, 39.1 18 obese controls, M/F 5/13, 44.1 17 obese BS patients, M/F 10/7, 39.4 22 healthy controls, M/F 9/13, 33.9	Gastric bypass	12 months	Inoculation of clinical specimens onto selective media (blood agar, chocolate agar, Sabouraud dextrose agar) and identification with MALDI-TOF MS (crevicular fluid)	After surgery and weight loss, the mean germ count increased, but not significantly. *Candida albicans* and *non-albicans Candida* species appeared after surgery; *Neisseria* was either absent throughout or eliminated after surgery.
Džunková et al., 2020 Cohort study [6]	Czech Republic	35, M/F 18/17, 48.0	Sleeve gastrectomy, Roux-en-Y gastric bypass, Omega loop gastric bypass, laparoscopic gastric plication	12 months	16S rRNA gene sequencing (saliva)	Increased proportion of *Veillonella species* after the decrease of BMI. *Streptococcus oralis* had a positive correlation with BMI. *Megasphaera micronuciformis* proportion increased when the BMI decreased.
Hashizume et al., 2015 Cohort study [24]	Brazil	27, M/F 1/26, 45.0	Roux-en-Y gastric bypass	6 months	Inoculation of clinical specimens onto selective media (Mitis salivarius bacitracin Agar, Rogosa SL agar, Sabouraud dextrose agar with chloramphenicol) and identification based on colony morphology and biochemical tests (saliva)	Salivary levels of *Streptococcus mutans* increased following BS.
Kim et al., 2025 Case-control study [23]	Republic of Korea	55 total, M/F 55/0, 36.0 31 obese BS patients, M/F 31/0, 37.0 24 lean controls, M/F 24/0, 35.0	Sleeve gastrectomy	6 months	16S rRNA gene sequencing (subgingival plaque, saliva, and oral swab)	Distinct species associated with periodontal disease found in the obese, surgically treated group in subgingival plaque (*Filifactor alocis, Peptostreptococcaceae* spp., *Prevotella* spp., and *Treponema maltophilum*). Microbiomes associated with a healthy state increased over time (*Streptococcus salivarius* and various *Veillonella* spp.). Clusters containing periodontal pathogens, including *Porphyromonas* spp., tended to diminish.
Ribeiro et al., 2023 Case-control study [26]	Brazil	40 total, 20 obese BS patients, M/F 5/15, 34.9 20 obese controls, M/F 5/15, 31.7	Roux-en-Y gastric bypass	6 months	16S rRNA gene sequencing (saliva)	Both interventions changed in different degrees the salivary inflammatory biomarkers and microbiota but did not improve the periodontal status after 6 months.
Sales-Peres et al., 2015 Cohort study [25]	Brazil	50, M/F 8/42, 38.9	Roux-en-Y gastric bypass	12 months	RTq-PCR (crevicular fluid)	*Porphyromonas gingivalis* increased after BS.
Shillitoe et al., 2012 Cohort study [22]	USA	29 M/F 7/22, 41.0	Roux-en-Y gastric bypass	12 weeks	RTq-PCR (saliva)	No changes in the levels of bacteria that exceeded 2-fold, except for the *Bifidobacteria* species, which showed a 2.4-fold increase in patients without DM type-2 and a 10-fold increase in DM patients. The levels of circulating endotoxin and TNF-α had decreased.
Stefura et al., 2022 Cohort study [27]	Poland	45 M/F 18/27, 43.5	Sleeve gastrectomy, Roux-en-Y gastric bypass	6 months	16S rRNA gene sequencing (oral swab)	Bacteria from phylum *Bacteroidetes* increased in abundance in the oral cavity 6 months after BS. Patients achieving at least 50% of excess weight loss presented similar results to the entire study group. Patients with less favorable outcomes presented an increase in the phylum *Fusobacteria* and a decrease in the phylum *Firmicutes* in the oral cavity.

## Data Availability

All the data presented in this review and supporting its conclusions are available upon request from the corresponding author.

## Supplementary Materials

The following supporting information can be downloaded at: https://www.mdpi.com/article/10.3390/antibiotics14070695/s1. Supplement S1. Search strategy.
