# Supplementary material for: Potential Shifts in the Oral Microbiome Induced by Bariatric Surgery—A Scoping Review
Source: Antibiotics (Basel). 2025 Jul 10;14(7):695. doi: 10.3390/antibiotics14070695 (PMC12291853; doi:10.3390/antibiotics14070695)
Supplement: Supplementary file 1 [file antibiotics-14-00695-s001.zip › antibiotics-3724852-supplementary.pdf]

Suppl S1. Search strategy

Database	Search formula	Number of records
Pubmed/ Medline	**(bariatric surgery) AND (oral microbiome OR oral microbiota OR oral bacteria)** ("bariatric surgery"[MeSH Terms] OR ("bariatric"[All Fields] AND "surgery"[All Fields]) OR "bariatric surgery"[All Fields]) AND ((("mouth"[MeSH Terms] OR "mouth"[All Fields] OR "oral"[All Fields]) AND ("microbiome s"[All Fields] OR "microbiomic"[All Fields] OR "microbiomics"[All Fields] OR "microbiota"[MeSH Terms] OR "microbiota"[All Fields] OR "microbiome"[All Fields] OR "microbiomes"[All Fields])) OR (("mouth"[MeSH Terms] OR "mouth"[All Fields] OR "oral"[All Fields]) AND ("microbiota"[MeSH Terms] OR "microbiota"[All Fields] OR "microbiotas"[All Fields] OR "microbiota s"[All Fields] OR "microbiotae"[All Fields])) OR (("mouth"[MeSH Terms] OR "mouth"[All Fields] OR "oral"[All Fields]) AND ("bacteria s"[All Fields] OR "bacteriae"[All Fields] OR "bacterias"[All Fields] OR "microbiology"[MeSH Subheading] OR "microbiology"[All Fields] OR "bacteria"[All Fields] OR "bacteria"[MeSH Terms])))	80
Web of Science	#1 bariatric surgery (All Fields) and oral microbiome (All Fields)<br>#2 bariatric surgery (All Fields) and oral microbiota (All Fields)<br>#3 bariatric surgery (All Fields) and oral microbiome (All Fields)	107
Cochrane Library	#1MeSH descriptor: [Bariatrics] explode all trees<br>#2bariatric[a]<br>#3MeSH descriptor: [Bariatric Surgery] explode all trees<br>#4MeSH descriptor: [Gastroplasty] explode all trees<br>#5MeSH descriptor: [Jejunoileal Bypass] explode all trees<br>#6MeSH descriptor: [Gastric Bypass] explode all trees<br>#7"Sleeve gastrectomy"<br>#8"Weight Loss Surgery"<br>#9"duodenal-jejunal bypass"<br>#10"gastrojejunostomy"<br>#11DJB<br>#12RYGB<br>#13MeSH descriptor: [Saliva] explode all trees<br>#14saliva[a]<br>#15Oral<br>#16MeSH descriptor: [Mouth] explode all trees<br>#17MeSH descriptor: [Periodontium] explode all trees<br>#18MeSH descriptor: [Periodontal Ligament] explode all trees<br>#19MeSH descriptor: [Gingiva] explode all trees<br>#20MeSH descriptor: [Gingival Crevicular Fluid] explode all trees<br>#21GCF<br>#22MeSH descriptor: [Microbiota] explode all trees<br>#23Microbiome<br>#24Microflora<br>#25Microbial<br>#26MeSH descriptor: [Microbiology] explode all trees<br>#27microbio[a]<br>#28MeSH descriptor: [Mycobiome] explode all trees<br>#29MeSH descriptor: [Bacteria] explode all trees<br>#30MeSH descriptor: [Fungi] explode all trees<br>#31#1 OR #2 OR #3 OR #4 OR #5 OR #6 OR #7 OR #8 OR #9 OR #10 OR #11 OR #12<br>#32#13 OR #14 OR #15 OR #16 OR #17 OR #18 OR #19 OR #20 OR #21<br>#33#22 OR #23 OR #24 OR #25 OR #26 OR #27 OR #28 OR #29 OR #30<br>#34#31 AND #32 AND #33	11
